# Accuracy of the ABFW ‒ Screening for speech disorders in early childhood

**DOI:** 10.1016/j.bjorl.2025.101697

**Published:** 2025-08-20

**Authors:** Marianna Momoe Nanakuma Matsumoto, Bruna Fernanda Alves da Silva, Maria Luiza Paulo de Oliveira Costa, Amanda Aparecida Carneiro, Daniela Regina Molini-Avejonas

**Affiliations:** Fundação Faculdade de Medicina da Universidade de São Paulo (FMUSP), Departamento de Fonoaudiologia, São Paulo, SP, Brazil

**Keywords:** American speech-language-hearing association, Diagnostic screening programs, Primary health care, Child, Accuracy

## Abstract

**Objective:**

To verify the accuracy of a protocol for the Brazilian population of the ABFW ‒ Adapted Child Language Test, named ABFW Triagem.

**Methods:**

This was a diagnostic accuracy study carried out at the speech therapy outpatient clinic of one of Brazil's leading public universities. Through the database, protocols were selected for patients aged between 0 and 5 years and 11-months who visited the outpatient clinic between 2016 and 2019 with any speech and hearing complaint. A follow up was carried out in 2024 to find out whether the speech therapy hypothesis was assertive or not.

**Results:**

188 protocols were selected and only 43 caregivers answered the researcher's call. The ABFW Screening protocol showed 72.1% accuracy and 64.3% balanced accuracy. Of all the hypotheses, the main one was language alteration characteristic of autism spectrum disorder (88.89%) with a sensitivity of 94.12% and specificity of 92.31%.

**Conclusion:**

The ABFW protocol adapted for speech and hearing screening proved to be a tool with the potential to make significant contributions to Brazilian public policies of interest to Primary Health Care. We suggest a greater volume of other speech therapy diagnostic hypotheses so that it can be validated and used by specialists, thus fulfilling the methodological rigor and development essential for its quality.

**Level of evidence:**

Is this diagnostic or monitoring test accurate? (Diagnosis) ‒ Level 2 (Individual cross-sectional studies with consistently applied reference standard and blinding).

## Highlights

• 188 protocols were analyzed.
• Balanced accuracy of 64.3% for all speech therapy hypotheses.
• Sensitivity of 94.12% and specificity of 92.31% for the diagnostic hypothesis of AS.

## Introduction

In 2023, a survey by the *Ministério da Saúde* in conjunction with the Maria Cecilia Souto Vidigal Foundation and the *Instituto de Saúde* revealed that 12% of Brazilian children up to the age of five have suspected developmental delays and do not display the behaviors and skills expected for this age group. The areas studied were motor, cognitive, language and socioemotional skills. In addition, a previous study showed that the incidence of delay is greater among the most socially vulnerable families.^1^ In 2022, Europe launched a pocket book titled “Primary health care for children and adolescents”, with Sustainable Development Goals that go beyond under-five mortality, now examining the social determinants of health problems and improving well-being.^2^^,^^3^

Looking at early childhood goes beyond child survival but toward a holistic view of the health of children and adolescents and shifts in attention toward health promotion, disease prevention, early management of risk factors and monitoring of chronic conditions.^4^

Primary Health Care (PHC) is the user's main point of entry; it acts as a coordinator and organizer of care for health care networks, offering longitudinal care to the population under its health responsibility. Early identification in PHC through screening allows children to have access to specific behavioral interventions that improve long-term outcomes.^5^^,^^6^ Effective screening requires not only a theoretical basis but also clinical experience and the correct and accurate use of screening protocols, which make it possible to arrive at the most reliable diagnostic hypotheses based on scientific evidence and not just deductions and uncertainties.

Child development screening should be part of the health professional's routine within PHC, as it enables not only early detection and intervention but also health promotion and prevention actions, with quality of life as the main commitment, so that children can have healthy growth and development.^7^^,^^8^

With respect to neurodevelopment, speech and language are two of the main domains, along with gross and fine motor skills, personal and social skills, cognition and activities of daily living.^9^ In Brazil, the main professional who works directly in these domains is the speech therapist, in addition to working in areas such as orofacial motricity, cognition, voice and hearing.^10^^,^^11^

Universal screening in early childhood is a challenge, given the cultural, socioeconomic and contextual factors that make children a varied group and difficult to assess with a simple screening tool.^12^ In this sense, this research sought to verify the accuracy of a protocol for the Brazilian population that was adapted by Samelli et al. (2014),^13^ named the ABFW ‒ Infant Language Test, resulting in speech therapy Diagnostic Hypotheses (DHs).

## Methods

### Ethical considerations

This study was approved by the Research Ethics Committee of the University of São Paulo (USP) - USP School of Medicine (FMUSP), number 2.437.351 and CAAE 80664717.4.0000.0065, and all participants signed the Informed Consent Form (ICF).

### Study design

This was a cross-sectional study of diagnostic accuracy that was carried out in two stages. In the first stage, the speech-language pathology and audiology Diagnostic Hypothesis (DH) of the children assessed at the Primary Health Care Research Laboratory (LIF APS) were identified. In the second stage, the guardians were contacted to check whether the hypothesis had been confirmed.

### Location of research

LIF APS was a speech therapy screening laboratory run by the Speech Therapy course at FMUSP, which closed in 2022. At the time, patients could be referred via hospitals or basic health units in the region, other locations or by spontaneous demand.

### Participants

We initially found 188 subjects aged 0–5 years and 11 months who had been to LIF APS from 2016 to 2019. The families sought care for children with complaints of altered language development, speech, hearing, orofacial motor skills or voice.

Inclusion criteria: Children up to 5 years and 11 months of age; Children who have been screened in the laboratory; Acceptance of the TCL; ABFW Screening complete.

Exclusion Criteria: No success in contacting the person responsible for follow-up.

### Instruments and materials

#### ABFW ‒ child language test

The ABFW is a Brazilian protocol that is considered a reference, as it covers and provides a reliable range that serves as a basis for comparison with normality in child development, made up of subtests that assess different areas involved in the communication process: phonology, vocabulary, fluency and pragmatics. It is designed to assess children from 2 to 12 years of age.

#### ABFW ‒ screening

Parents/guardians answer the screening protocol, and the results are analyzed by the applicator. It is a version adapted by Samelli et al. (2014) from the ABFW ‒ Infant Language Test protocol, containing objective and descriptive answers in the areas of audiology, fluency, phonology, orofacial motor skills, vocabulary, pragmatics, voice and interaction. At the end, the applicator fills in a summary table according to the answers provided by the parents/guardians with the options “Inadequate” or “Adequate”; if the answer “Inadequate” is greater than 50%, it is considered “Compromised” in that particular area analyzed. At the end, undergraduate students and supervising speech therapists analyze and arrive at a speech therapy DH.

### Data collection

Once the inclusion and exclusion criteria had been met, the researcher contacted all the family members, asking them what their final diagnosis was after a full assessment, or the course of action suggested after screening. They were also asked whether or not they were receiving care following the referrals given by LIF APS, and if not, whether they would like to receive care. The families were contacted by telephone throughout 2024, and a standard text was drawn up for contacting all the families: “Hello, this is ____, a postgraduate speech therapy student at FMUSP. I'd like to speak to the person responsible for ____. I saw that your child was seen on __of ___of 201_ at the Speech Therapy Screening. At the time, he/she was diagnosed with ____. I would like to know how you are today, whether the diagnosis has remained the same or has changed. I'd also like to know if you're in therapy, if so, where, and if not, if you'd like to see me”. Three attempts were made to call you during each period (morning, afternoon and day).

All the data collected were tabulated in an Excel spreadsheet by the researcher. In the first stage, the technical procedure used for data collection was indirect documentation, i.e., analysis of the protocols completed by parents/guardians at the time of ABFW-Screening at LIF APS application from 2016 to 2019. Based on the results of the ABFW ‒ screening summary table, the DHs were standardized according to Molini-Avejonas (2015) to include alterations in language, alterations in language characteristic of neurological problems, alterations in language characteristic of autism spectrum disorder, alterations in language characteristic of syndrome, language disorder due to hearing loss, alterations in the orofacial myofunctional system, alterations in voice, phonological disorders, alterations in reading and writing, alterations in fluency, more than one diagnosis, and no alterations in speech-language or hearing. In the second stage, telephone contact was made with the 188 families in the first quarter of 2024 to confirm whether the speech therapy DH had in fact become a diagnosis or not, as well as to check whether the children were being monitored by a speech therapist after the screening was carried out at the LIF APS.

### Statistical analysis

Analysis of the accuracy of ABFW ‒ Screening was carried out using balanced accuracy, which is not influenced by unbalanced classes, i.e., if one speech therapy DH stands out from the others. The volume, accuracy, specificity and sensitivity of each speech therapy DH were analyzed.

## Results

**Fig. 1:**
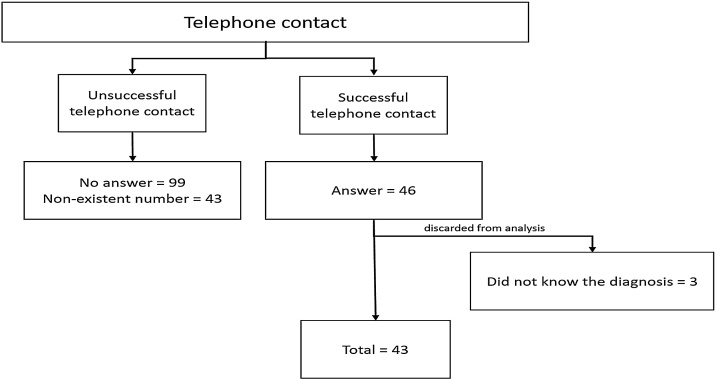
Telephone contact.

After the exclusion and inclusion criteria, only 43 subjects were actually used for analysis. Fig. 1 shows the sample flowchart.

**Fig. 2:**
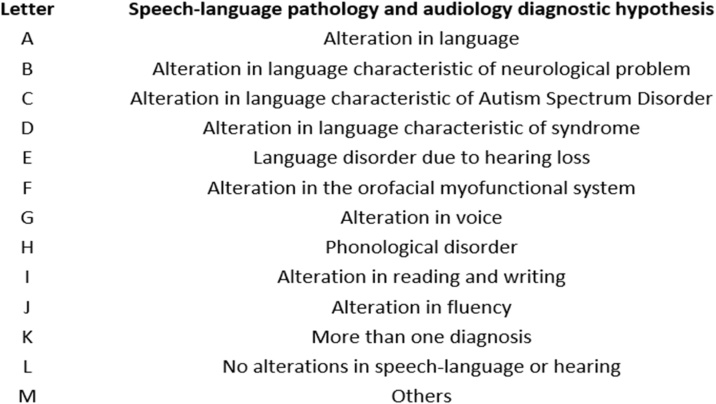
Speech-language pathology and audiology diagnostic hypothesis.

Fig. 2 shows how the terminology used for speech therapy DH was standardized for this study.

Table 1 shows the volume of speech-language pathology and audiology DHs, four of which stand out: A (language disorder), C (language disorder characteristic of autism spectrum disorder), H (phonological disorder) and K (more than one diagnostic hypothesis).

**Table 1:** Volume of speech-language pathology and audiology diagnostic hypothesis.

DH	N	% Total
A	2	4.65
B	3	6.98
C	17	39.53
D	3	6.98
E	1	2.33
F	3	6.98
G	0	0.00
H	5	11.63
I	0	0.00
J	5	11.63
K	0	0.00
L	4	9.30
M	0	0.00
Total	43	100%

DH, Diagnostic Hypothesis; N, Number of subjects.

**Fig. 3:**
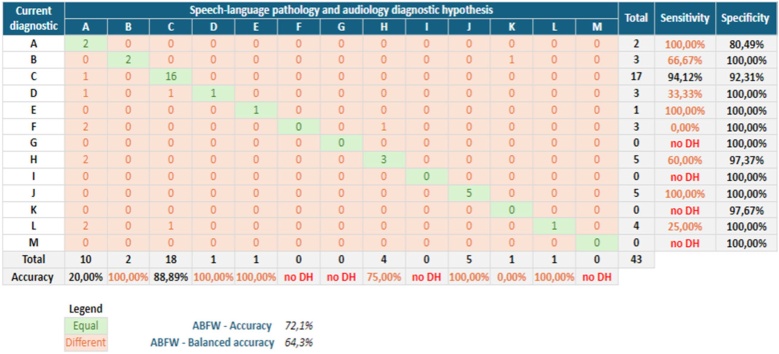
Statistical analysis.

After the researcher contacted the parents/guardians to collect the child's current diagnosis, the main results were a balanced accuracy of the ABFW screening protocol (64.3%), an accuracy of the letter C DH (88.89%), a sensitivity of 94.12% and a specificity of 92.31%. Fig. 3 shows the general statistical analysis of the ABFW and for each speech therapy DH. The only analysis possible was for DH letter C due to its volume; the other data should be analyzed with caution.

**Fig. 4:**
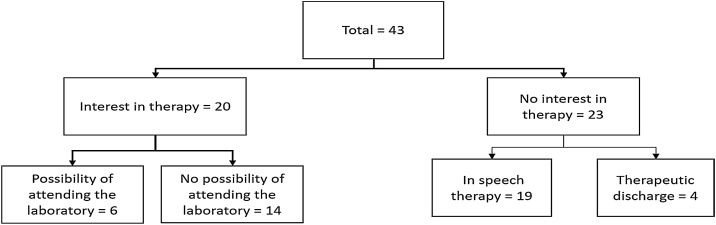
Outcome of referrals.

As for the outcome of the referrals, Fig. 4 shows the outcome of the 43 subjects. Of these, 20 parents/guardians were still interested in speech therapy for their children, while the other 23 families had been discharged or were undergoing speech therapy following a referral from LIF APS.

## Discussion

The ABFW protocol is considered a reference, as it covers and provides a reliable range that serves as a basis for comparison with normality in child development in the areas of fluency, phonology, vocabulary and pragmatics.^14^ In this study, ABFW screening was used in an adapted form, as applied in the LIF APS, with four additional areas, namely, audiology, voice, orofacial motor skills and interaction.

It is important to note that in Brazil, speech therapy encompasses areas beyond speech, language and hearing. According to the Federal Council of Speech Therapy (2024).^15^

The speech therapist is a health professional with a full degree in speech and hearing therapy who works autonomously and independently in the public and private sectors. They are responsible for health promotion, prevention, assessment and diagnosis, guidance, therapy (habilitation and rehabilitation) and improvement of the speech therapy aspects of peripheral and central auditory function, vestibular function, oral and written language, voice, fluency, speech articulation and the myofunctional, orofacial, cervical and swallowing systems. He also carries out teaching, research and administrative activities.

In this study, 188 early childhood subjects were analyzed, covering the age range from 0 to 5 -years and 11-months. The speech-language pathology and audiology diagnostic hypotheses that stood out the most were C (language alteration characteristic of autism spectrum disorder), A (language alteration), H (phonological disorder) and K (more than one diagnostic hypothesis).

DH letter C suggests that the main diagnosis is Autism Spectrum Disorder (ASD), accounting for 38% of the total cases. It was also the only hypothesis that could be analyzed in its entirety, considering accuracy, sensitivity and specificity. This finding corroborates the findings of international studies showing that in one sample, 69%–87% of participants had structural language difficulties, and 33%–45% reached the clinical thresholds for behavioral difficulties with pragmatic deficits.^16^ One of the main explanations for hypothesis C being in evidence may be the increase in population awareness and public health response globally, changes in case definitions that span diagnostic boundaries over time, increased diagnosis of milder forms and increased identification of autism in previously underdiagnosed cases.^17^

The second DH suggested that the main diagnosis could be language delay or Developmental Language Disorder (DLD), representing 27% of the total. This finding corroborates the literature, which states that despite its high prevalence and significant impact, DLD is a relatively unknown disorder.^16^ In the article by Bishop (2010)^18^ entitled “Which neurodevelopmental disorders are researched and why?” Only 0.13 articles per 100 individuals affected by DLD in the UK population were found for the years 2000‒2009. In the article by Saul (2023),^16^ using the same metrics and analysis as the previous author, only 0.16 publications were found for the years 2010–2019 for every 100 children affected.

The third DH suggested that Speech Sound Disorder (SSD) was the main diagnosis, accounting for 11% of the total sample. Its low volume can be explained by the age range of the subjects. According to national articles, the age with the greatest number of participants whose complaints were related to phonology was 5-years.^19^^,^^20^

The accuracy was balanced, as the DH letter C had a greater effect than the other letters. As a result, the accuracy was 64.3%. This result is understandable given that only 43 people in charge answered the calls and responded to the researcher. This result is in line with one of the most widely used triage tools in hospital emergency departments, known as the Manchester Triage System, where national studies point to its accuracy and easy applicability.^21^ One of the studies investigated the accuracy of this system in hospital emergencies with 400 patients and obtained an accuracy of 68.8%.^22^ According to the articles, knowledge about the quality of accuracy makes it possible to obtain good judgment and more assertive decision-making and thus obtain the desired results.^23^^,^^24^ Low accuracy can lead to wasted time and energy, as well as patient and family dissatisfaction.^25^ This is not desired or expected in the application of speech therapy screening.

This study highlights the importance of screening as a pass/fail tool, which is fast and low cost. In this study, data from subjects with alterations was analyzed in depth. It is important to emphasize that the ABFW Screening protocol also requires clinical reasoning from health professionals, especially speech therapists, and that it alone is not a universal screening protocol.

Despite some limitations of this study, such as the number of responses to the researcher's calls, the results obtained allowed us to reflect not only on the importance of greater investment in research to detect possible delays in a child's development at an early stage to make monitoring and intervention effective but also on how to provide ongoing treatment for these children, given that only 53.5% had been discharged or were undergoing speech therapy

## Conclusion

This study achieved its objective by verifying the accuracy of ABFW screening. On the other hand, it is important to mention that with a larger number of participants, the results obtained would be more generalizable (although it would have been possible to carry out a statistical analysis with the current number of children in at least one DH).

It is extremely important that the results presented here contribute to valid and standardized screening instruments. However, it is also important to bear in mind that there may not be a single instrument capable of universal screening, given the multiple variables to be considered.

The importance of structured screening also became clear, as in addition to the application of protocols, we have continuity of treatment for the children we treat. Applying screening without the prospect of treatment for the child and support for the family goes against one of the basic principles of the Brazilian health system, known as SUS in portuguese “Sistema Único de Saúde”, the INTEGRALITY of the subject, which neglects all their needs. It is crucial to rethink screening and its continuity since, according to the literature, delays in diagnosis and treatment can lead to emotional overload for children and their families, increased parental anxiety, possible side effects of treatments, and additional difficulties in the emotional and social development of children and their families.

From this perspective, it is essential to invest in more research aimed at addressing the integrality of the child and the family, training parents to be aware of developmental milestones and promoting appropriate and timely interventions.

## ORCID ID

Marianna Momoe Nanakuma Matsumoto: 0000-0001-5577-2559

Bruna Fernanda Alves da Silva: 0009-0006-7015-2888

Maria Luiza Paulo de Oliveira Costa: 0009-0006-9514-4040

Amanda Aparecida Carneiro: 0000-0001-7495-3814

Daniela Regina Molini-Avejonas: 0000-0002-9768-882X

## Funding

This study was financed in part by the Coordenação de Aperfeiçoamento de Pessoal de Nível Superior – Brasil (CAPES) – Finance Code 001.

## Declaration of competing interest

The authors declare no conflicts of interest.
